# Connectotyping: Model Based Fingerprinting of the Functional Connectome

**DOI:** 10.1371/journal.pone.0111048

**Published:** 2014-11-11

**Authors:** Oscar Miranda-Dominguez, Brian D. Mills, Samuel D. Carpenter, Kathleen A. Grant, Christopher D. Kroenke, Joel T. Nigg, Damien A. Fair

**Affiliations:** 1 Department of Behavioral Neuroscience, Oregon Health & Science University, Portland, Oregon, United States of America; 2 Advanced Imaging Research Center, Oregon Health and Science University, Portland, Oregon, United States of America; 3 Division of Neuroscience, Oregon National Primate Research Center, Beaverton, Oregon, United States of America; 4 Department of Psychiatry, Oregon Health & Science University, Portland, Oregon, United States of America; Wake Forest School of Medicine, United States of America

## Abstract

A better characterization of how an individual’s brain is functionally organized will likely bring dramatic advances to many fields of study. Here we show a model-based approach toward characterizing resting state functional connectivity MRI (rs-fcMRI) that is capable of identifying a so-called “connectotype”, or functional fingerprint in individual participants. The approach rests on a simple linear model that proposes the activity of a given brain region can be described by the weighted sum of its functional neighboring regions. The resulting coefficients correspond to a personalized model-based connectivity matrix that is capable of predicting the timeseries of each subject. Importantly, the model itself is subject specific and has the ability to predict an individual at a later date using a limited number of non-sequential frames. While we show that there is a significant amount of shared variance between models across subjects, the model’s ability to discriminate an individual is driven by unique connections in higher order control regions in frontal and parietal cortices. Furthermore, we show that the connectotype is present in non-human primates as well, highlighting the translational potential of the approach.

## Introduction

It is now largely recognized that while the endeavor is vast, understanding brain organization and dynamics will be critical for the improved well-being of our society. From technological advancements [1] to improved health outcomes, a better characterization of this organ is likely to be advantageous to several fields of study. Particularly valuable will be understanding variation– not simply variation in brain organization across different populations, but also *between individuals*.

Studies of the brain and in particular those that utilize functional neuroimaging, have historically largely focused on the shared variance across large populations. These studies often neglect the vast heterogeneity that exists in both typically developing and atypical populations (see [2]). Similar to the Human Genome Project, which has brought unprecedented progress in science, technology, and medicine through the sequencing of an individual’s genome, identifying and characterizing the unique and individualized functional architecture (*i.e.* the connectotype) of the human brain may have parallel utility.

The primary goal in the current report was to determine whether resting-state functional connectivity is capable of identifying a so-called “functional fingerprint,” in individuals (*i.e.*, a specified model of the whole brain specific to a single person –see methods). In particular, we examined if the proposed methodology is A) able to predict the activity of a given ROI simply by knowing the activity of all of the other brain regions at a given point in time, B) if so, determine if the generated models act as a unique (fMRI-derived) brain signature which is specific to an individual (*i.e.* similar to a genotype), and C) to determine what connections and brain regions are most highly variable across subjects (*i.e.* which connections drive the ability to distinguish an individual).

Resting state functional connectivity MRI (rs-fcMRI) measures correlate spontaneous brain activity between brain regions while subjects are at “rest” – not performing a goal directed task. The approach in the current manuscript deviates from more traditional measurements of functional connectivity [3] and follows several basic steps to generate a simple linear mathematical model of an individual. The idea is based on the principle that the activity in any given ROI can be predicted by the weighted sum of the activity of its functional neighboring regions (*i.e.* all other brain regions). While alternative approaches have been proposed elsewhere [4]–[13], here we propose and show that 1) this individualized model is highly reliable and has the ability to classify an individual at a later date using a limited number of non-sequential frames (defined as a volume of the corresponding BOLD signal from all the ROIs used in a given parcellation at each sampling time *i.e*. TR), 2) the observed phenomena is present not only in humans but in non-human primates, highlighting the preserved network dynamics across species and the translational potential the approach, and 3) that brain regions and connections important for higher order processing (*i.e*., frontal-parietal systems) are critically important for determining the connectotype. Just as unique information within a patient’s genome can govern how an investigator or even a clinician approaches an individual, we hope that the connectotype will be equally informative with this regard in time.

## Methods

### Subjects

Experiments were performed in data acquired from 27 healthy adult humans (16 females) age 19 to 35 years, and 11 adult male macaques.

### Humans

All procedures were approved by the Institutional Review Board at Oregon Health and Science University. Participants were recruited through advertisements in the community, such as posted flyers, online ads and on Oregon Health and Science University’s clinical trials website. Potential participants were screened on the phone for initial eligibility. Exclusion criteria included a history of neurological trauma, a head injury with loss of consciousness, a medical condition which could affect cognition, or current substance abuse. Participants were also excluded for having a current depressive or manic episode, a history of psychosis, bipolar disorder, learning disability, ADHD, current substance addiction, or for taking long acting psychoactive medication. Informed consent was obtained for all participants.

Participants had to have normal use of both hands, had to be right-handed and had to have normal or corrected-to-normal vision and normal hearing. Participants were also excluded if they had any contraindications to undergoing an MRI scan, such as pregnancy, metal in the body or a history of claustrophobia. Eligible participants were then scheduled for an initial visit. At this visit participants completed two semi-structured clinical interviews, 3 modules from the Kiddie Schedule for Affective Disorders and Schizophrenia [14] modified for adults to assess child disorders, and one from the Schedule for Affective Disorders and Schizophrenia [15] modified for DSM-IV and the Structured Clinical Interview for DSM Disorders [16]. Participants were excluded if those interviews revealed any of the psychiatric exclusion criteria outlined above.

Participants also completed an brief intelligence test (WASI, Wechsler Abbreviated Scale of Intelligence, [17] and an achievement test (WRAT-4, Wide Range Achievement Test, [18]). These measures were used to assess IQ, as well as to identify potential learning disabilities. Participants were excluded if they had an estimated full scale IQ under 85 or a suspected learning disability, as indicated by a difference of more than 1.5 standard deviations between cognitive functioning and achievement scores (as obtained in WASI and WRAT).

At their MRI visit participants were screened for substance abuse with a urine toxicology screen administered at the beginning of the visit. The toxicology screen included tetrahydrocannabinol (THC), cocaine, opioids, amphetamine and methamphetamine. Participants were excluded from completing the MRI scan if they tested positive for cocaine, opioids, amphetamines or methamphetamines, but not if they tested positive for THC.

#### Group H27

The human sample included 27 healthy subjects. The average age was 27±4 years with a minimum age of 19 and a maximum age of 35. Sixteen subjects were females.

#### Group H5

This group comprises a subset of 5 individuals from group H27. Each participant had 510 or more frames (after motion correction) on a second scan acquired 1 week after the first. This sample was used to validate the model’s ability to make individual predictions of a subject’s identity on a subsequent scan (out-of sample data). The number of frames for each participant is listed in Table 1.

**Table 1:** Frames in group H5.

	Data after movement correction			
	First scan		Second scan	
Subject	Frames	Time (min)	Frames	Time (min)
1	589	19.63	544	18.13
2	565	18.83	544	18.13
3	565	18.83	314	10.47
4	522	17.40	447	14.90
5	514	17.13	548	18.27

### Macaques - Group M11

Eleven cynomolgus macaques were also examined (all adults, ages 5.4±0.40, min 4.8, max. 5.9 yo), and were chosen to have no common parents or grandparents, from the pedigreed Oregon National Primate Research Center breeding colony. For approximately 6 months prior to imaging, each monkey was individually housed in a stainless steel cage measuring 1.6×0.8×0.8 *m* (Allentown Caging, Allentown, NJ, USA) in a vivarium with 12 h light/dark cycle (with lights on at 7 am) that was maintained at 21±1 °C and 30–50% humidity. Each animal had visual, auditory, and olfactory access to other monkeys in the vivarium, and limited physical access to a neighboring monkey. The monkeys were fed a diet of fresh fruit and 1 *g* banana-flavored pellets in quantities sufficient to maintain a positive caloric intake.

All animal procedures were conducted in accordance with the “Guidelines of the Committee on the Care and Use of Laboratory Animal Resources” (National Health Council, Department of Health, Education, and Welfare, ISBN 0-309-05377-3, revised 1996). Prior to their implementation, procedures were approved by the Institutional Animal Care and Use Committee (IACUC) of the Oregon National Primate Research Center as they were in compliance with all local, state, and national regulations pertaining to the humane use of animal subjects.

No animals were sacrificed to obtain the data in this paper. Environmental enrichment is provided to all non-human primates in the protocol in terms of each animal had visual, auditory, and olfactory access to other monkeys in the vivarium. Monkeys are provided either pair housing with another monkey for 2 hours each day or tactile contact with a neighboring monkey through a grooming partition in the housing cage. Finally, all monkeys are provided with enriching toys, mirrors, foraging materials and the ability to control their meal patterns through the operant panel in the housing cage.

### Imaging acquisition and processing

#### Human MRI data acquisition

All MRI scans were performed on a Siemens 3 Tesla TIM-TRIO system. Structural images were obtained using a sagittal magnetization-prepared rapid gradient echo (MP-RAGE) three-dimensional T1-weighted sequence (TR = 2.3 *s*, TE = 3.58 *s*, flip angle = 10°, TI = 900 *ms*, voxel size = 1×1×1 *mm*, slices = 160). Functional images were obtained using a gradient-echo, echo-planar sequence sensitive to blood oxygen level-dependent (BOLD) contrast (TR = 2000 *ms*, TE = 30 *ms*; FOV = 240 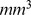; flip angle = 90°; 3.75×3.75×3.8 *mm*). Full brain coverage was obtained with 33 contiguous interleaved 3.8 *mm* axial slices acquired parallel to the plane transecting the anterior and posterior commissure. Steady state magnetization was assumed after four frames (∼8 seconds).

For the resting-state fMRI, participants completed either two scans consisting of 150 acquisitions (5 participants) or one scan of 600 acquisitions (22 participants). A longer scan was introduced in order to insure that sufficient volumes would be retained for data analysis after removal of volumes identified as having excessive movement.

#### Human image processing

Functional data preprocessing involved several steps. Each dataset was corrected for odd *vs*. even slice intensity differences attributable to interleaved acquisition without gaps. Head movements were first corrected by re-aligning all volumes to the middle volume of the first run using a six parameter motion-correction algorithm within and across runs. Intensity normalization was applied to each run to a whole brain mode value gradient of 1000. Atlas transformation into Talairach coordinate system of the functional data was computed for each individual via the MP-RAGE, and then each run was resampled in atlas space on an isotropic 3 *mm* volume combining the six parameters for movement correction and atlas transformation in one interpolation. As an additional step, the frame-by-frame spatial deviations of the acquisition time-series were assessed using the temporal derivative of the time courses [19], [20].

Several additional preprocessing steps were used for the rs-fcMRI data to reduce spurious variance unlikely to reflect neuronal activity. These steps included: (1) a temporal band-pass filter (0.009 *Hz* < *f*<0.080 *Hz*) and spatial smoothing (6 *mm* full width at half maximum), (2) regression of six parameters obtained by rigid body head motion correction, (3) regression of the whole brain signal averaged over the whole brain, (4) regression of ventricular signal averaged from ventricular ROI, and (5) regression of white matter signal averaged from white matter ROI. Regression of first order derivative terms for the whole brain, ventricular, and white matter signals were also included in the preprocessing. Analyses were also conducted without the whole brain regression.

#### Macaque MRI scanning

Imaging was performed during a single session for each animal subject on a 3T Siemens Tim Trio scanner with a 15-channel knee coil adapted for monkey head scanning. Subjects were sedated with an initial dose of ketamine (5 *mg/k*g), intubated, and maintained under 1% isoflurane anesthesia for the duration of MRI procedures. Physiological monitoring throughout anesthesia included heart rate, respiration, and peripheral oxygen saturation. Data acquisition included four high-resolution T2-weighted structural images (TR = 3200 *ms*, TE = 497 *ms*; 0.5 *mm* ^2^ in plane resolution, 1 *mm* slice thickness, 56 slices, FOV = 128×128 *mm*), which were averaged to improve signal-to-noise ratio. A functional MRI scan lasting 30 minutes was then begun exactly 45 minutes after the time of ketamine administration (delaying the beginning of the acquisition as necessary to maintain the time since ketamine induction across all animals), using a gradient echo echo-planar imaging (EPI) sequence sensitive to BOLD contrast (TR = 2070 *ms*, TE = 25 *ms*, FA = 90*°*, 1.5×1.5×1.5 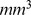 voxels, 32 slices with interleaved acquisition, FOV = 96×96 *mm*). A fieldmap scan was acquired (TR = 450 *ms*, TE = 5.19 *ms*/7.65 *ms*, FA = 60°, 1.25×1.25×2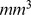 voxels, 40 slices, FOV = 120×120 *mm*) to correct for image distortion (see below).

#### Macaque Image Processing

The raw fMRI data underwent standard fMRI preprocessing using the FMRIB software library’s FEAT preprocessing tools. This included slice-timing correction, rigid-body correction for head motion, unwarping of fieldmap distortions, and rigid-body co-registration of the fMRI volumes with the high-resolution T2-weighted structural image. The structural image was transformed using 12-parameter affine registration to conform to a T2-weighted atlas image, which was an average of 112 monkeys (http://brainmap.wisc.edu/monkey.html). This atlas image was also linearly registered onto the widely-used macaque F99 atlas, freely available as part of the CARET software package (http://brainvis.wustl.edu/wiki/index.php/Main_Page). Thus, the registration parameters obtained from each step allowed raw fMRI images to be transformed into F99 space, combining motion correction, fieldmap unwarping, and atlas transformation in one interpolation step. As with the human data, several additional steps were also taken to prepare the data for connectivity analyses [21], including temporal bandpass filtering (0.009 Hz < f<0.080 Hz), spatial smoothing (3 mm full-width at half-maximum), and regression of nuisance signals. The latter included the whole-brain signal and the six parameters related to rigid-body motion correction. Analyses were also conducted without the whole brain regression.

### Parcellations

Four ROI atlases were used, all of which were based on histological studies done in the macaque and deformed to fit homologous areas on the human brain using methods that have been validated in prior studies [22]–[24]. These atlases are the Fellemen and Van Essen (FVE) [25], Lewis and Van Essen (LVE) [26], Paxinos [27], and the Markov atlases [28], each with a variable number of regions. Paxinos atlas = 351 ROIs, Markov = 184 ROIs, LVE = 176 ROIs, FVE = 156 ROIs. The four cortical parcellations were deformed from the macaque to the human PALS atlas using the validated deformation mapping algorithm [29], [30]. Time series were computed for cortical regions of interest (ROIs) by averaging the signal intensity across all voxels within a given ROI at each time point.

Human-Macaque Registration: ROI examination across species was conducted utilizing surface based atlas registration using the CARET software package. The process uses a spherical, landmark-based registration algorithm [29], [30]. Landmarks for registration included a standard set of regions that are likely to be homologous across species including visual areas V1, V2, MT, and frontal eye fields, primary auditory cortex, olfactory, gustatory, somatosensory, and primary motor cortex [24], [30], [31]. Differences in overall cortical shape are minimized by mapping each cortical surface to a standard configuration (*i.e.,* a sphere), then, each sphere is registered to one another constrained by this set of homologous landmarks. Deformation from macaque to human cortex results in a large non-uniform expansion of parietal, temporal, and frontal cortex, and much less expansion in presumably conserved regions between species (*i.e*. V1, motor cortex, etc). Landmark-based registration provides a powerful method for analyzing structural and functional organization between humans and macaques [23], [32], [33]. Each of the four cortical parcellations were deformed from the macaque to the human PALS atlas using the deformation mapping provided by the above procedures.

**Figure 1:**
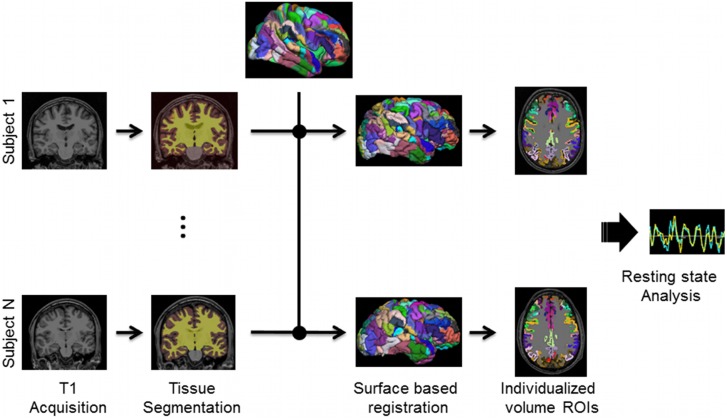
Individualized Markov ROIs. For each T1-weighted MP-RAGE volume, we individually segmented each brain after individual registration to the fsaverage surface using Freesurfer. An individual model was calculated in the 20 subjects of whom individualized ROI generation was possible (see methods).

We conducted the analyses with parcellated ROIs in two ways. Analyses were initially conducted on the atlas based ROIs, which does not account for possible variations in individual neuroanatomy. Therefore, we also registered one of our atlases to each individual subject via surface registration (see Figure 1). This process is outlined in the following subsection.

#### Individualized Markov Parcellation

First, the Markov atlas was transformed from the PALS surface to the fsaverage surface (FreeSurfer average) via the deformation maps provided by the Surface Management System Database and WebCaret Online Visualization (http://sumsdb.wustl.edu/sums/index.jsp). Then, each subject’s T1-weighted MP-RAGE underwent the ‘recon-all’ segmentation procedure from the Freesurfer software (http://surfer.nmr.mgh.harvard.edu). One of the many outputs is a gray matter surface and gray matter volume, specific to each subjects’ anatomy. Using Freesurfer surface to surface registration tools, each segmented grey matter volume was converted to a 3D surface and registered to the fsaverage surface. These mappings were then used to register the Markov parcellation atlas to each individual’s surface anatomy. The resulting subject defined regions were transformed from each subject’s individual gray matter surface to 1 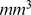 volume space using a gray matter mask to assign the voxels. The individualized regions were then transformed from 1 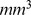
native space to 3 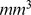 Talaraich space to match the atlas registered volume data for each individual. Regions on the order of 1 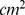 or less could not be accurately accounted for in a 3 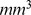 isotropic volume in all subjects, likely due to individual variability in cortical folding and gray matter thickness. Thus, a total of seven subjects were excluded as they had one or more regions fail this transformation step. The resulting regions, individualized to each subject’s gray matter, were used to create time courses from the processed BOLD data (see Figure 1).

### Software

A variety of software packages were used for image processing: These included in-house 4dfp software from Washington University (http://nrg.wikispaces.com/), FSL (http://fsl.fmrib.ox.ac.uk/fsl/fslwiki/), and freesurfer (http://surfer.nmr.mgh.harvard.edu/). Areal region of interest (ROI) deformations were done as part of the freely available CARET software package http://brainvis.wustl.edu/wiki/index.php/Main_Page). Surface ROIs for each area were converted to volume using caret software (3 *mm* thickness in humans and 1.5 *mm* thickness in macaques). Deformation maps from the Surface Management System Database and WebCaret Online Visualization (http://sumsdb.wustl.edu/sums/index.jsp). Registrations were then carried out using FSL’s linear (flirt) and nonlinear (fnirt) registration tools. Tissue segmentation into white and gray matter was performed on the T1 image using Freesurfer software. All other analyses were calculated using Matlab.

### Connectotyping

In brief, the model was constructed by the following general procedures. First, in order to eliminate spurious correlations, autocorrelations were removed from all timecourses. Next, to obtain the model, each ROI residual was modeled as the weighted sum of all the other ROIs residuals in the same frame. Such modeling results in an underdetermined system which we chose to solve using the pseudoinverse (for comparison) and truncated singular value decomposition. Testing the strength of the model occurred using several approaches: timeseries prediction, in-sample and out-of-sample individual classification, and prediction.

#### Removing autocorrelations

Autocorrelations that prevailed after filtering (preprocessing) were removed from all the time courses in order to eliminate spurious correlations. Those autocorrelations are always present even in the absence of evoked changes in neuronal activity due to the dispersive nature of the response at each voxel [34]. Autocorrelations could also be related to slight changes in the local magnetic field due to scanner instabilities [35]. To remove autocorrelations, a linear model was fit to predict each ROI’s time course as the weighted sum of its historical values:



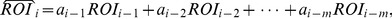
(1)where 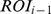 represents the normalized bold signal of a given ROI at the time *i*. 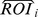 is the estimated activity of that given ROI at the time *i* (as predicted by the model), *m* indicates the historical values included in the model, and 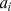 are the weights (fits) used in the prediction at each time *i*.

Different values of *m* were tested. At the end, we set 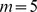, that is a balanced number between the predictions and the number of included parameters.

Then, the residuals were calculated by subtracting the prediction from the actual activity for each ROI:



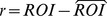
(2)


On average, the correlation coefficient between the predicted and actual ROI’s activity was 0.8540 with a standard deviation of 0.0513. The residuals were used to obtain a functional connectome model for each subject.

#### Obtaining the model

**Figure 2:**
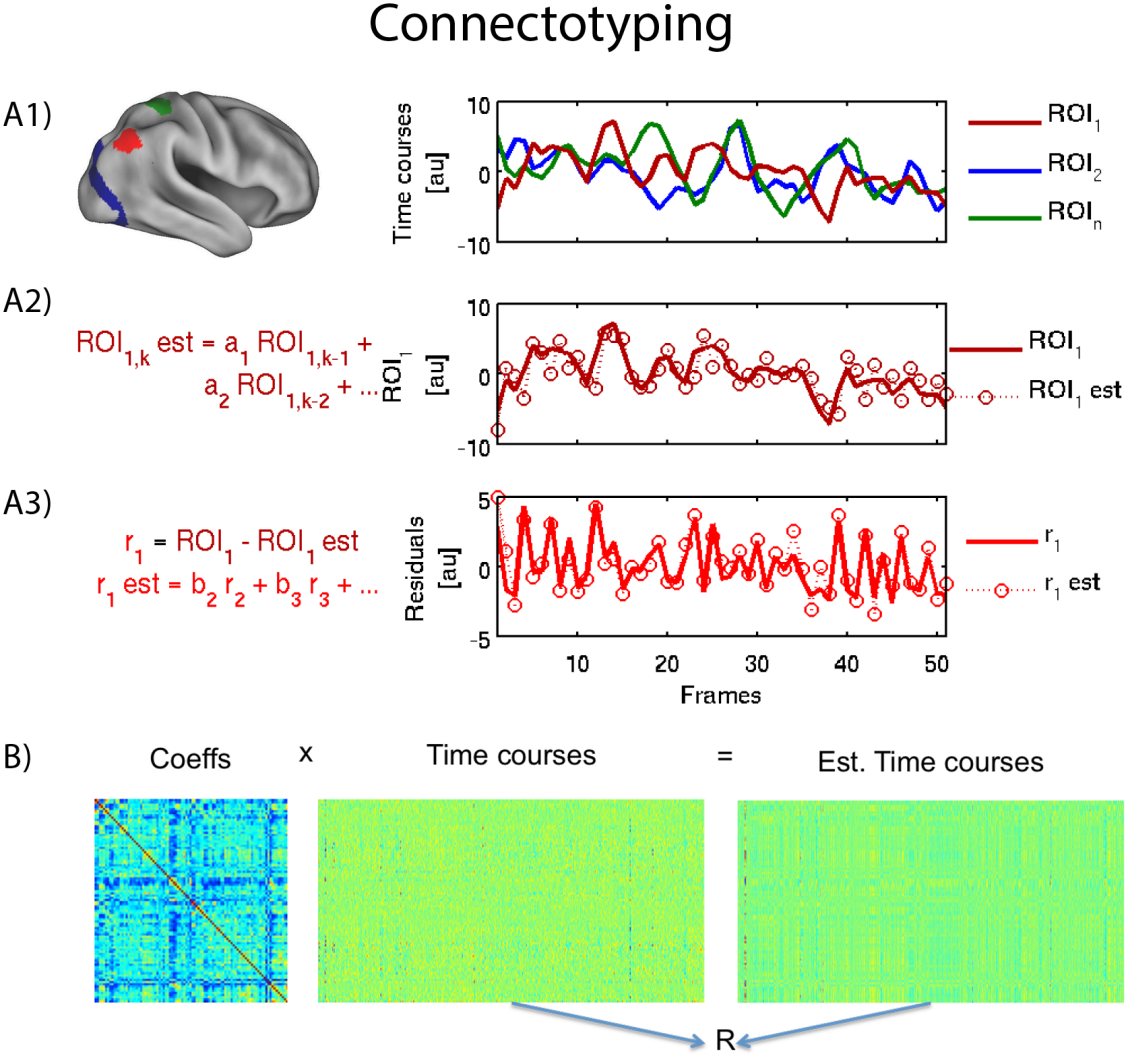
Connectotyping: Once the time courses are created for each ROI (panel A1), a mathematical model is fit to predict the activity of each ROI given its historical values (panel A2). Then, the estimations are subtracted from the original time courses. Next, each ROI’s residual is modeled as the weighted sum of the residuals of all other ROIs (panel A3). Finally, all the coefficients are grouped in a matrix, or “the model”. Each subject’s timecourses can be used to make an estimation of the actual values (panel B1) and the fit can be quantified by the correlation coefficient of measured and estimated timecourses. This approach allows us to test the model in the same subject the model was obtained from or using a different subject (panel B2).

For each parcellation and processing protocol, the residual’s ensemble was modeled as the weighted sum of all the others residuals, as shown in the following (Note a brief synopsis of our approach is provided in Figure 2):
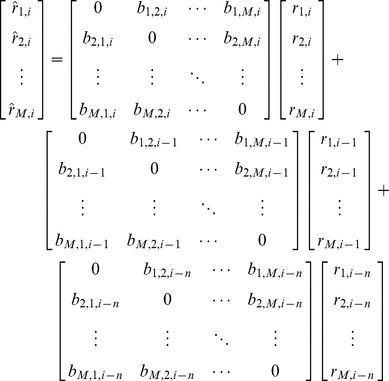
(3)where *M* is the number of ROIs for each parcellation, 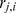 is the residual of the ROI *j* (this index can go from 1 to *M*) at the time *i*, 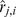 is its estimation, and 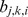 is the optimized coefficient used to predict the ROÍs residual *j* at the time *i* from the residual *k* at the time *i* (both, *i* and *j* goes from 1 to *M*). *n* indicates how many historical values are included in the prediction.

Condensed version of equation 3 is



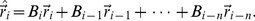
(4)Here, 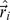 is a vector that concatenates the expected values from all the required ROIs in a given parcellation at time *i*. 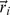 is the ensemble residuals at time *i* that were obtained from the previous analysis and *B* is a directed matrix of coefficients. Here, *n* also refers to the historical information used in the prediction. Setting 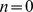 makes 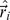 depend only on concurrent information, while increasing *n* adds historical information about the neighbors in the prediction. In this study we did not include historical information, *i.e*. we set 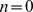. Future studies may include historical information, then *n* will need to be adjusted according to the required number of frames.

Equation 4 can be condensed again as



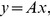
(5)where 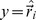, and *A* and *x* are the reordered *B*’s and 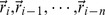 considered in the calculation of *y*. From here, the problem consists on solving the equation 5 for *A*. *A* can be solved by calculating *x’*s pseudo-inverse or by regularization.

#### Pseudo-inverse (PINV)

From linear algebra, 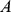 can be solved as



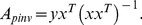
(6)While the pseudo-inverse method renders the solution with the lowest discrepancy between the predictions and measurements (in a least squares sense), its use often leads to over-fitting and its predictions are highly susceptible to noise on ill-posed systems [2]. The coefficients calculated in ill-posed systems tend to change in sign and have a large norm. Thus, it provides a higher bound estimate but cannot be relied on without cross validation.

#### Regularization (TSVD)

There are alternative approaches to solve equation 5 robustly, which means that instead of expecting the maximum match between predictions and measurements, the solution is a compromise between predictive power and another criteria, such as robustness in regards to misestimating parameters or maximizing predictions in out-of-sample data.

In this paper, we used the truncated singular value decomposition method (tsvd). In brief, from equation 5, *x* is factorized into its singular value decomposition (svd), which is the product of 3 matrices. One of the matrices represents the singular values of *x*. The singular values indicate, among other things, how susceptible to noise the system is. Then, by cancelling (truncating) some of the more problematic singular values, *x* is recalculated using its more stable singular values and then the system (equation 5) is solved (for a detailed explanation see [36], [37]). One of the problems with this method is that sometimes it is difficult to decide the cutoff to pass/cancel singular values.

The svd of *x* is given by



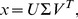
(7)Where *U* and *V* are matrices that contain the left and right singular vectors of *x.* 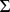 is a diagonal matrix that contains the singular values of *x* sorted decreasing monotonically
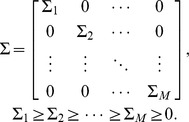



The smaller singular values of *x* are more susceptible to noise. In general, setting those singular values to zero makes the system more robust to noise at the expense of predictive power. If some singular values are set equal to zero, an estimation of *x* can be recalculated by setting to zero some of its singular values and then applying equation 7 using a truncated 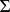, so 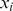 is the truncated version of *x* where all its singular values greater than *i* were set to zero. This truncated version of *x* can be plugged into 6 to find an estimation of A 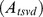.

The more singular values are set to zero, the lower the norm of the resulting coefficients (insensitive to noise) but also the larger discrepancy between predictions and measurements.

To estimate the optimal number of singular values to be used in the truncation, we used two methods: first, we maximized predictions in fresh, out-of sample data. The second method consists on finding stable norms of the residuals and estimations. Both of these approaches are described in more detail below.

#### Maximizing predictions

In this approach, we used two sets of data from an individual. We used the residuals of the first data set to calculate *x* and all its possible truncations. Then, we used the second data set to fill the vector *y*. All the possible 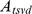
’s were calculated using the entire set of truncated versions of *x* in equation 6. The values of *y* were used as *x* in equation 5 and finally the correlation coefficients for each truncation were calculated. The optimal truncation value was selected as the one that provided the maximum correlation coefficient between the predictions and the residuals in the fresh data.

#### Finding stable norms of the residuals and estimations

This approach just requires one data set. Again, all the possible truncated solutions were calculated as described in the previous method. Then, the predictions were calculated for each truncated solution. We calculated the norm of the solution and the norm of the residuals for each truncation and we selected as optimal value, the value at which the rate of change of the norms are both at a maximum of 1% for a given frame (see Figure S1 in File S1). This tsvd method can be implemented using just set of data from a subject. However, the correlation coefficient between predictions and measurements can be lower than the ones obtained by the use of the previous described method to determine the number of singular values to truncate.

## Results

The current work outlines an approach for constructing a functional connectivity model of an individual’s connectome. Conceptually, each individual’s model is obtained by predicting the activity of each node based on the concurrent activity of all the other regions after the removal of autocorrelations. Such modeling results in an underdetermined system which we chose to solve using two commonly used methods, truncated singular value decomposition (tsvd) as well as the pseudo inverse. The methodology is illustrated in Figure 2.

### Timeseries prediction

**Figure 3:**
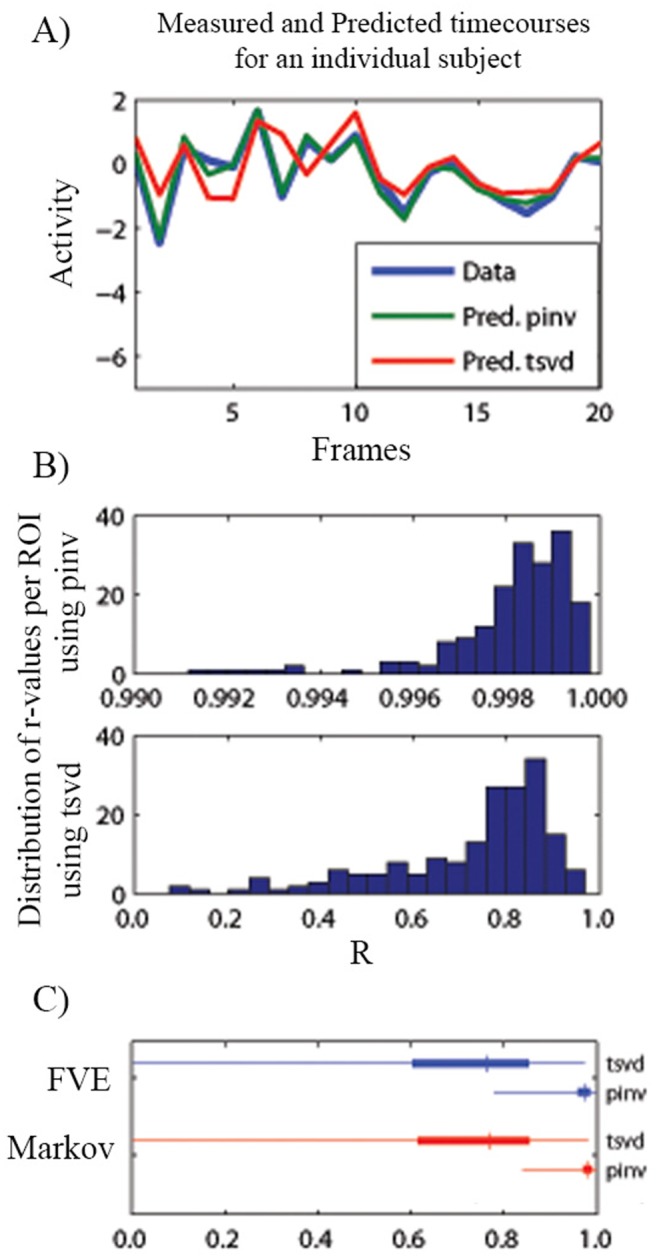
Modeling results. Panel A shows the measured and predicted timecourse using both the pinv and tsvd methods of the ROI L-V1 for the first subject of group H27 (20 frames are shown from the Markov parcellation). Panel B shows the distribution of the correlation coefficients between the predicted and measured ROIs calculated by the pinv and tsvd methods, respectively. Panels C shows the distribution of correlation coefficients between predictions and measured time courses for 27 subjects, using the FVE and Markov parcellations, pinv and tsvd-based predictions are shown on each panel. Thin lines indicate the range of values, thick lines correspond to the 25 to 75 percentiles and the vertical marker is the median of each distribution. Both methods show a strong ability to predict the timeseries of a given ROI within an individual.

Bold activity prediction was first conducted on 27 adult participants (group: H27). To assist in direct comparability to the analysis with non-human primates all analyses were conducted on four whole brain ROI parcellations stemming from well-known areal macaque atlases. These atlases included the Felleman and Van Essen (FVE) [25], Lewis and Van Essen (LVE) [26], Paxinos [27], and the Markov atlas [28], deformed to fit a common human atlas (see methods). For each combination of these 4 parcellations, two models were obtained for each subject: one model by the pseudo-inverse (pinv) and the second by truncated single value decomposition (tsvd) (see methods). From here, on “fresh” data, ROI’s activity was predicted for each model (*i.e.* participant) using equation 5. On average, the correlation coefficient between the predicted and measured time courses was 0.9823 with a standard deviation of 0.0188 for the pinv method, while the corresponding mean and standard deviation for the tsvd were 0.7692 and 0.1750, respectively. An example timeseries, the distribution of r-values, and distinct results for the FVE and Markov parcellations are shown in Figure 3 (for remaining parcellations see Figure S2 in File S1). Both the pinv and tsvd show highly accurate prediction of the timeseries of a given ROI (Note: despite the higher correlation for the pinv, as noted in methods, the TSVD score is the more robust estimate, less prone to over fitting, and provides more stability, as shown and described below).

### In-sample and out-of-sample individual classification

In this section we describe how strongly our functional connectome (individually obtained functional connectivity model, or “connectotype’) is able to describe or predict the individual subject it was created on versus other individuals. We begin this effort by exploring the models ability to predict an individual from “fresh” data within the same scanning session (in-sample predictions) using group H27. We follow this experiment by examining the models ability to predict an individual based on “fresh” data in a scanning session one week later on 5 adult participants, group H5 (Out-of-sample predictions). The experiment was performed using all ROI parcellations (as above). In both scenarios (*i.e*. in–sample and out-of-sample) our models were capable of predicting an individual with high precision (described below). Importantly, these analyses were replicated in a population of non-human primates.

#### Pseudo inverse (H27 group)

By randomly selecting 45% of the frames from each participant, a model was obtained by the pseudo inverse method for each one of the 27 subjects. We then applied the model to 15 percent of fresh data (*i.e.,* making sure that the frames used here were not used when each model was obtained), which was used to predict the corresponding time courses of these fresh frames. The correlation coefficient between the predicted and measured time courses was used to classify whether the data comes from the same subject or not. Panel a) on Figure S3 in File S1 compares the distribution of correlation coefficients obtained when the data to obtain model and to make the predictions comes from the same subject *versus* the distribution when the data comes from the other subjects. The correlation coefficients quartiles 1 and 3 are 0.70 and 0.77 when data to obtain the model and make predictions comes from the same participant while the same quartiles’ correlation coefficients are 0.40 and 0.50 when data comes from different participants. The difference between self and others was highly significant (p<0.0001), but note the high variance induced in “other” condition using pinv.

#### TSVD (H27 group)

To generate our model using TSVD we again randomly selected 45% of the frames from each participant to obtain a model. However, for TSVD, as opposed to pinv, regularization is required prior to testing. Thus, for each subject 15% of the frames (randomly selected) from each one of the 27 participants were used to regularize the model by maximixing the predictions of this fresh data (see methods). The resulting model then was used to predict a new fresh set of 15% of the frames from each participant (again, frames were randomly selected making sure that no frames were repeated at any stage of the analysis). This approach leads to several ways to test the model. To determine the effect of regularization, we grouped the possibilities into 4 cases:

Case 1 (*itself*): model, regularization, and prediction were performed in the same subject.
Case 2 (*itself regularized in others*): model and prediction were performed in the same subject, but regularization (4) was performed in a different subject.
Case 3 (*fooled by regularization*): the regularization and prediction were performed in the same subject, but the model was obtained from a different subject.
Case 4 (*others*): all the remaining possibilities.

Experimentally, each case was statistically distinct (panel b on Figure S3 in File S1). The highest correlation coefficient between predictions and measurements is achieved when the model, regularization, and prediction were performed in the same subject (∼0.84) – *i.e*., Case 1. Regularizing the model in a different subject decreases the predictive power (from 0.84 to 0.81) – *i.e.* Case 2. The case “fooled by regularization” reports an average correlation coefficient of 0.68 (*i.e*. Case 3), while the remaining combinations average a correlation coefficient of 0.62 (*i.e*. Case 4).

The difference in the average correlation coefficient between the cases “itself” and “itself regularized in others” roughly corresponds to what is gained by regularization when tsvd is compared to pinv. *Fooled by regularization* indicates the potential lost in predictive power (compared to pinv) when the regularization is not done properly. Finally, *others* is the control case that quantifies the variance that is shared by all the subjects. These findings suggest that unique individual patterns for a given model lead to a strong prediction. The results also indicate that there is a significant amount of shared variance across participants.

### Out-of sample classification - Group H5

This analysis was conducted on five human subjects. The following subsections describe the results of the experiment when data from the same participants obtained from two scanning sessions elapsed by one week are used to obtain the model and make the predictions, respectively.

#### Pseudo inverse (H5 group)

**Figure 4:**
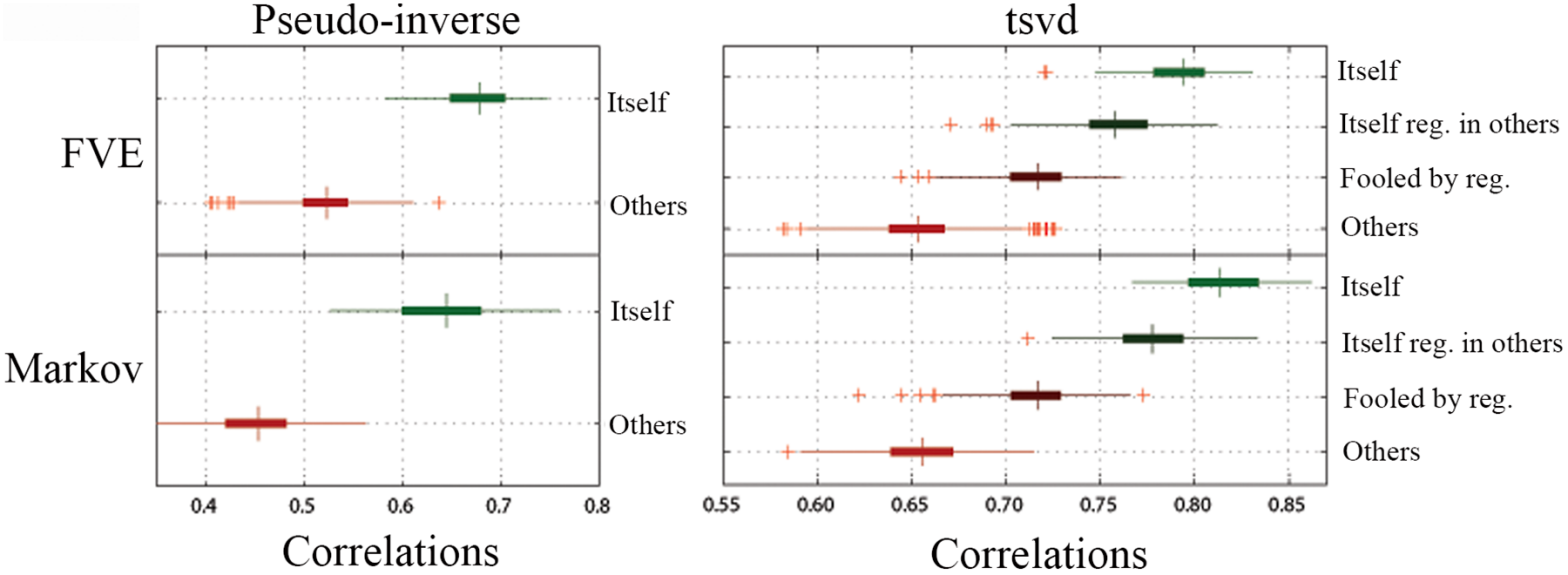
Predicting subjects in group H5. Model (pinv and tsvd) predicting same subject on second scan date for group H5 (*i.e.* predicting itself *versus* predicting others) for the FVE and Markov parcellations. Case "Itself" corresponds to the result when the model, regularization, and prediction were performed on the same subject. "Itself reg. in others" is the case when model and prediction were performed in the same subject, but regularization was performed in a different subject. "Fooled by regularization" comes from the comparison where the regularization and prediction were performed in the same subject, but the model was obtained from a different subject. The remaining combinations are concatenated in the case "Others". In all instances, the model performs more strongly in predicting self versus others; however, a significant amount of variance is accounted for in the “other” cases.

For the group H5, a functional connectotype was identified for each subject by randomly selecting 45% of the total frames from the first scan. Then, 15% of the frames from scan 2 (1 week later) were randomly selected and used to predict each subject. This experiment was repeated 10 times randomly selecting different frames on each realization. Across atlases, the obtained model using the pseudo inverse was able to reliably distinguish an individual *vs* other subjects on a second scan. The model explained approximately 65–70% of the variance in the same individual, where the model explained roughly 50% of the variance in other subjects (left side panels on Figure 4 and Figure S3 panel c in File S1).

#### TSVD (H5 group)

A model was obtained for each participant by randomly selecting 45% of the frames from the first scanning session. Then, for each subject, 15% of the frames (randomly selected) from the second scan were used to regularize the model by maximixing the predictions of this fresh data (see methods). The resulting model then was used to predict a new set of 15% of the frames from scan 2 (randomly selected and making sure that no one of the frames were repeated in the 2 datasets). This experiment was repeated 10 times resampling the frames. Again, we grouped all of the possibilities into 4 cases: *itself, itself regularized in others, fooled by regularization, others* (see description of cases in previous section).

For each one of the parcellations, the 4 cases were distinct (see right panels of Figure 4 and Figure S3 panel c in File S1). The highest correlation coefficient between predictions and measurements is achieved when the model, regularization and prediction were performed in the same subject (∼80%). Regularizing the model in a different subject decreases the predictive power.

**Figure 5:**
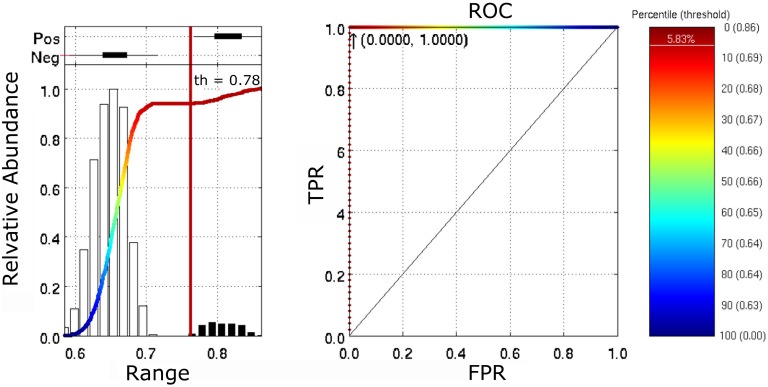
Classification power quantified by an ROC curve (using tsvd), group H5. The left panel shows the distributions (top) and the corresponding histograms (bottom) of the correlations of each subject predicting itself (Pos) and predicting others (Neg). The colored line is the cumulative distribution of correlations of the entire data set. The optimal classification is achieved when the threshold is set to 0.7825, which corresponds to the 5.83% of the largest correlation coefficients. This threshold leads to a true positive rate (TPR) of 1 and a false positive rate (FPR) of 0. As shown the classification accuracy is 100% with marked distinctions in the distributions. The performance here using tsvd was stronger than that of the pinv (Figure S4 in File S1). Analyses were performed using the Markov atlas.

We next used receiver operating characteristic (ROC) curves to quantify the classification power of the method for a given individual, where the average correlation coefficient between predicted and measured residuals was used as classification criteria. The optimal correlation coefficient was calculated as the one providing simultaneously the lowest false positive rate (FPR) and the maximum true positive rate (TPR), as shown in Figure 5 for the tsvd method and Figure S4 in File S1 for pinv.

### Prediction and classification using individualized ROIs

**Figure 6:**
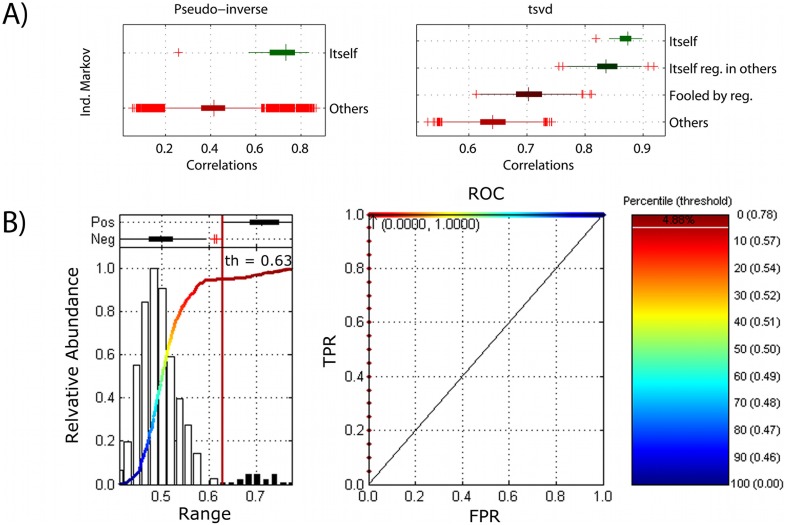
Predictions using individualized ROIs. A) This panel shows the average correlation coefficient when predicted and measured BOLD activity is compared. On the left, the results using the pinv method is used to calculate the model using 45% of the frames and predicting 15% of fresh in-sample data. On the right, indicates the 4 distinct groups when the tsvd method is used to predict data using 45% of frames to obtain a model, 15% of fresh in sample-data to select the number of singular values to preserve, and 15% of fresh in-sample data to predict bold activity according to the regularized model. B) This panel summarizes the predictive power using receiver operating characteristic curves (here, the singular values where calculated by finding stable norms of residuals and estimations, as described in methods).

To insure that the obtained results were driven by individual differences in functional connectivity rather than variation in anatomy (see methods), we calculated individualized anatomical based ROIs for each one of the human subjects included in this study (H27 group), as described in methods. We repeated the modeling to determine whether an individual connectivity matrix can predict with higher reliability fresh data from itself than data coming from others. Using the same sampling proportions (*i.e*., 45% to obtain the model, 15% to regularize, when required, and 15% to predict), we found that using individualized ROIs, the obtained model was able to predict BOLD activity similar to what was identified for the atlas based ROIs, as shown in Figure 6. Interestingly, tsvd renders, on average, a higher correlation coefficient when using individualized Markov ROIs to predict fresh data coming from the same subject (right panel on Figure 6A), compared to using the atlas based Markov ROIs (Figure S3, panel c in File S1). In these two cases, the truncation was selected by maximizing predictions of fresh data. These findings highlight that the model is not driven by individual differences in anatomy.

#### Predictions in macaques

**Figure 7:**
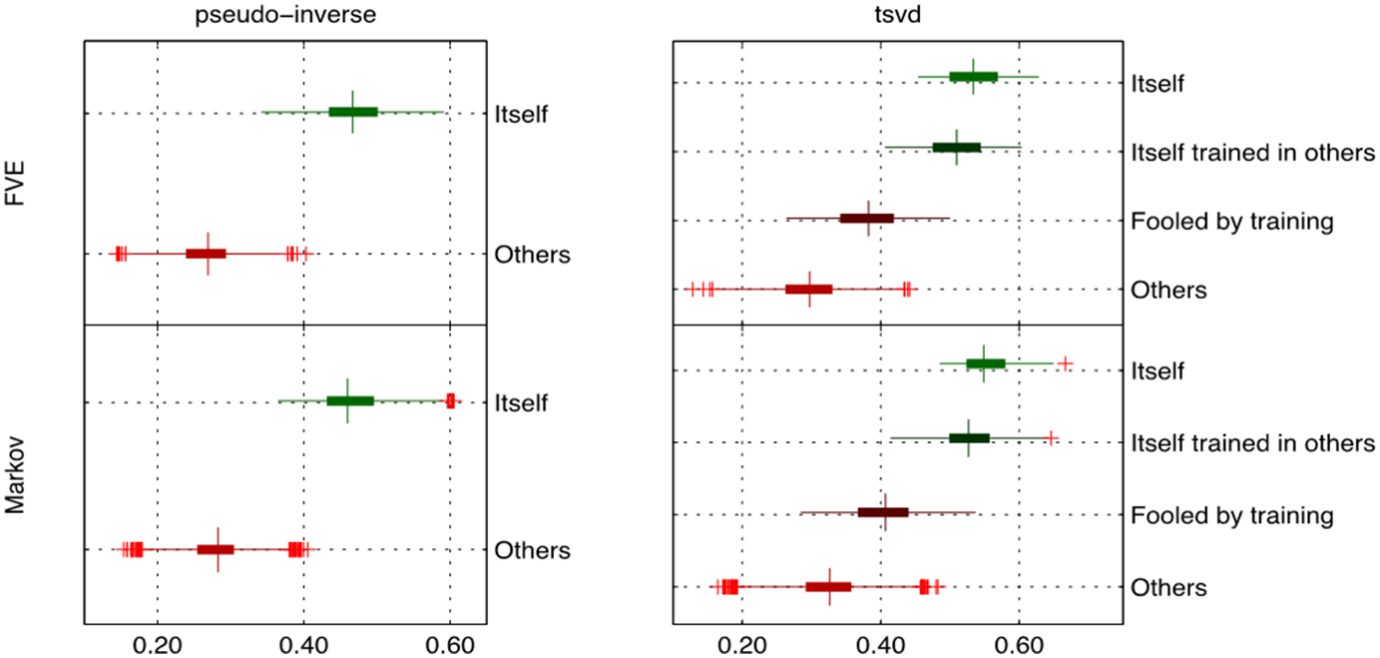
Predicting subjects in group M11 (11 Macaques). Distribution of average correlation coefficients for group M11 predicting itself *versus* predicting others. For tsvd, case 1 (*itself*): model, regularization, and prediction were performed in the same subject. Case 2 (*itself regularized in others*): model and prediction were performed in the same subject, but regularization (4) was performed in a different subject. Case 3 (*fooled by regularization*): the regularization and prediction were performed in the same subject, but the model was obtained from a different subject. Case 4 (*others*): all the remaining possibilities.

Importantly, because of the unconstrained nature of the resting state and other problematic issues with rs-fcMRI in humans (such as movement, and “free thinking”) [20], we also conducted our connectotyping experiments in a set of macaques under anesthesia. The experiments in macaques were performed as described for humans (in-sample, group H27). The samples used for model, regularization (if applicable, *i.e.* in tsvd), and prediction were different frames from the same-day scan. The variance explained is somewhat weaker to that obtained in humans; however, the model is still able to discriminate between *itself* and *others* and the tsvd improves the classification. The model obtained by the tsvd method was able to account for about 58% of the variance in the same subject where it accounted for only about 36% of the variance in other macaques (see Figure 7).

### Common versus variable features across participants

**Figure 8:**
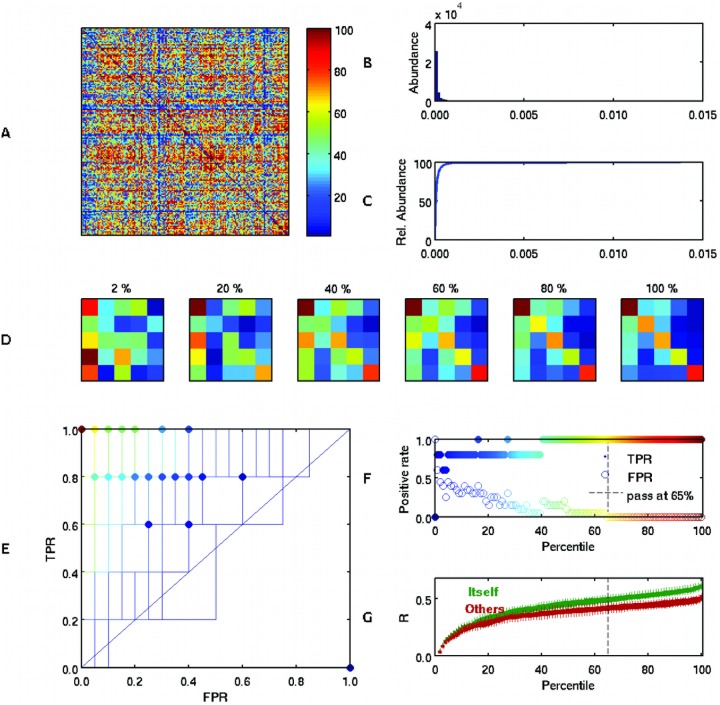
Predictions in the group of 5 human subjects (H5– second day scan) based on consistent or unique connection weights across the populations. After using tsvd to generate a model for each subject, the variance across participants was calculated for each entry (panel A). Panels B and C shows the distribution and cumulative distribution of the coefficient’s variance, respectively. From the cumulative distribution, the connections were grouped into 100 bins with similar variance (presented in panel A). Then, 100 masks were created setting to zero the connections with higher variance and incrementally using more preserved (lower variance) connections. The masks were applied to the first day model to predict the second day scan. Panel D shows the correlation coefficient between the predicted and actual time courses for each one of the subjects in the group H5. The cell *i, j* indicates that the model was obtained in the subject’s *i* first-day scan and that model was used to predict the subject’s *j* second-day time-courses. The sub-panel’s title indicates the percentile of most preserved connections (mask) used in the model. Panel E shows the 100 ROC (lines) and optimal threshold (dots) for each run (percentile). Panel F shows the optimal True Positive Rate (TPR) and False Positive Rate (FPR) as a function of the run. Panel G shows the distributions of correlation coefficients for all the subjects in group H5 predicting itself and predicting others, as a function of run. The dotted line indicates the point at which there is a clear separation between the two predictions. All of panels D-F highlight how the predictive nature of the model is dependent on the most variable connections across subjects. The most preserved (lowest variance) connections do little to distinguish between individuals.

While the connectotype of an individual is most predictive of that same individual, it is clear that any given model can account for much of the variance in other individuals. This suggests that the model structure for a given person is also highly overlapping with other individual model structures. Thus, we set out to determine what model based connections are typically most conserved across the population versus those that are more variable across the population. To determine the connections that are more preserved *versus* the more variable, we calculated a tsvd-based functional connectivity matrix for each one of the subjects in the H5 group. In this case, the tsvd model was obtained by finding stable norms of the residuals and estimations. Then, we calculated the variance across subjects for each one of the ROI x ROI connections. From the cumulative distribution of the variance, we incrementally selected the most consistent coefficients across subjects (lower variance) and set to zero the remaining connections for each model to make a mask. For each iteration we applied the resulting mask to each subject’s model. The resulting “masked” model was used to predict the second-day scan. Presumably the connections with lower variance across subjects should have a limited ability to distinguish between individuals. On the other hand when more variable links are added to the model, the ability to distinguish individuals should improve. Indeed, this is the case - shown when predicting second scan dates using both tsvd (Figure 8) and the pseudo inverse (Figure S5 in File S1). The phenomenon where regions of higher variance contribute more strongly to the self vs other prediction is also found when using fresh data on the set of 27 participants (see Figure S6 in File S1 and S7 in File S1).

**Figure 9:**
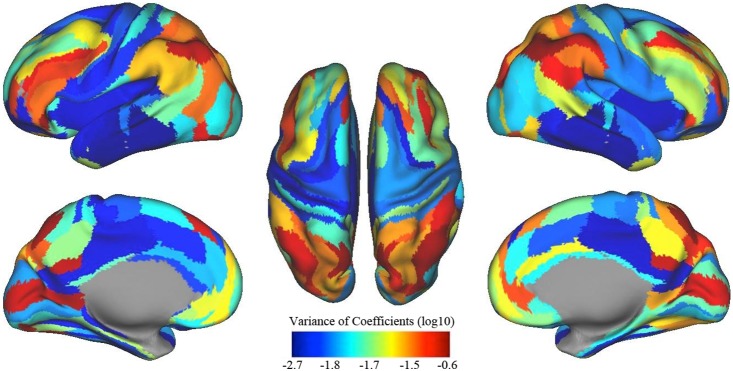
Shared *vs* individualized connections. The color indicates how stable the source connections are for each colored ROI. Color (in base 10 logarithmic scale) indicates the sum of the variance of the coefficients. The lower the value, the more stable (*i.e*., the more preserved) the connection across individuals (shown in the Markov parcellation).

Next, we used the variance matrix from group H27 to visualize on the brain, each connections variance across participants using the Markov parcellation. Figure 9 shows the sum of the variance *per* row (in log base 10), which is an indication of how stable the connections are to the targeted ROI. Notice that nodes of higher order task control systems in frontal and parietal cortices and areas along the midline are those with the highest variance among individuals.

### Required frames to make estimations

**Figure 10:**
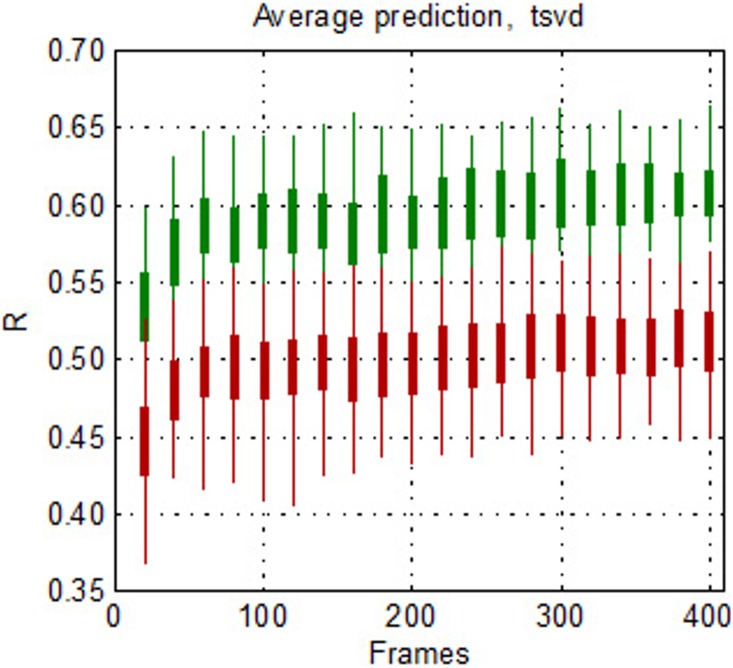
Frames required for prediction. The distribution of correlation coefficients for each model predicting itself (green) *vs* predicting others (red) is shown as a function of frames for the group H27. 60 frames produces stable self prediction, where below 60 frames the model is slightly reduced in its ability to predict an individual.

Next, we highlight yet another important benefit of this modeling approach. In contrast, to many functional connectivity methodologies, the methods proposed here do not require continuous timeseries data. Here we explored how much data is required to generate a stable model. To examine this, we used the group H27 to test how the predictions and the tsvd models reach their final value as a function of the number of frames included in the model. In this experiment, we sequentially increased the number of frames, starting with 20 randomly selected frames and incrementally increase the number of frames by 20 until a total of 400 frames were included in the model. On each iteration, the frames were selected randomly for each subject and a tsvd model was obtained. Then, the model was used to predict all the subjects in the group. This approach was repeated 10 times. As seen in Figure 10, robust prediction is obtained with between 40 and 60 frames. Surprisingly, between 60–100 frames seems to render estimations similar to the ones obtained when the entire data set is used. These results highlight the stability of the estimations with very limited amounts of data. The results also suggest that a strong and generally robust model for a given individual can be obtained with just over a minute of data.

### Preserved versus personalized connections

**Figure 11:**
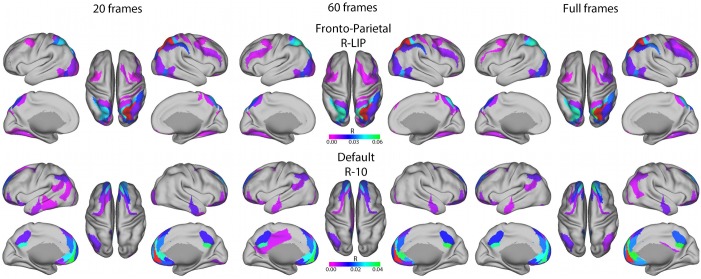
Group H27 resting state networks for 20, 60 and the entire number of frames used to calculate the model based tsvd connectivity map on the Markov atlas. A connection density of 20% (*i.e.* strongest 20% of coefficients) was used. Seed based analysis shows that fronto-parietal and default mode networks are well represented at the group level even down to 20 frames. Similar phenomena can be demonstrated in an individual subject (Figure S8 in File S1).

Finally, we assessed the ability of the connectotype model to recapitulate traditionally derived resting state networks (*i.e*., default, and fronto-parietal networks) at both the group (Figure 11, data visualized on the Markov parcellation) and individual subject level (Figure S8 in File S1). Default mode was derived by placing a seed in the anterior node of the default system (right hemisphere area 10) and the frontoparietal network by a seed in the parietal cortex (right hemisphere area LIP). We found that at the group level, the fronto-parietal and default mode networks are well represented with as little 20 frames of data. When choosing an individual subject at random, the default network is identified with 20 frames and above, but the fronto-parietal network is only partially represented at 60 and the full number of frames for this subject (Figure S8 in File S1). This may suggest that 60 frames is a sufficient amount of data for this method, but also highlights possible individual variability in traditional resting state networks. Overall, the resulting beta matrices obtained by this method are advantageous not only for their ability to ‘fingerprint’ an individual’s connectome but also in their ability to provide a biologically plausible connectivity profile with limited amounts of data (Data visualized on the Markov parcellation).

## Discussion

In the current report we show that the presented modeling approach on rs-fcMRI data is able to obtain a personalized connectivity profile for an individual. Importantly, the model, which can be obtained in less than two minutes of non-sequential resting state data, is able to reliably identify an individual on a subsequent scan with high precision. In addition, the proposed approach recapitulates established connectivity networks across the entire study population and in individuals. As a benefit to future translational and mechanistic studies, we show this phenomenon is not unique to humans but is also present in sedated non-human primates.

### Benefits of the current approach

The strength of the current approach lies in its simplicity. We propose a simple linear model to infer brain activity, where the activity of each ROI can be predicted as the weighted sum of all other ROIs (after removing spurious autocorrelations [34], [35] - sometimes overlooked in traditional fMRI studies). Removal of autocorrealtions is critical if historical information needs to be added. Autocorrelations render on average a correlation coefficient of 0.8540. When autocorrelations are removed, the obtained model is able to predict with high specificity residuals’ data coming from the same subject and also to find shared variance in the group. This phenomena is observed regardless of species or parcellations used in this study. We are aware that the brain does not process information linearly [38]; however, a completely accurate mathematical representation of functional interactions is, as of now, out of reach. Nonetheless the proposed model is capable of predicting with relatively high precision the activity of a given brain region at any given time point and the same individual upon a second scan.

PCA, ICA [6]–[9] and other multivariate methods [4], [5] have been used extensively to analyze fMRI, looking for individual and group biomarkers, and also to predict timeseries, having moderate to high levels of accuracy. These methods have also been used to examine test-retest reliability in these types of data [6]. While our method is not PCA or ICA, our approach is similar in that they pose the problem in terms of the linear 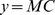, where 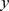 is the fMRI data and *M* and *C* correspond to the fMRI centered data and its eigenvectors in PCA or the weights and the data components in ICA, respectively. As this linear has no unique but infinite number of solutions, constrains needs to be added to solve the problem. Constrains and assumptions such as these make the different flavors of PCA and ICA. In our case, we assume that we can predict the ROI’s activity on each time point by the weighted contribution of the remaining ROIs. Next, we solved the resulting set of linear equations by regularization in order to minimize the effect of noise and overfitting. This modeling approach can be obtained with limited amounts of data and is able to account for a most of the observed variance in fresh in-sample and out-of-sample data, rendering it a strong approach to characterize individual connectomes.

A few recent studies have examined the ability of various functional connectivity models to predict particular aspects of brain activity [4], [39], [40]. While none of these efforts have reliably demonstrated an ability to identify an individual subject based on a given model, they all point out that such modeling can be complicated, in part because the number of brain regions to be modeled is much greater than the number of observations. This circumstance results in an underdetermined system of linear equations which are unable to be solved uniquely. However, this problem can be solved with various methods including dimensionality reduction [34], [41], frameworks such as statistical learning theory [39], feature selection, or regularization based algorithms [4], [40], [42]. Here we chose to solve the problem using the pinv and regularization methods, *i.e*. tsvd [36]; however, other frameworks may be equally informative with regard to generating individualized models.

### Regional variation that drives an individual’s connectotype

We note that a given individual’s model was not completely unique relative to other individuals. In other words, large portions of variance from another subjects scan could be explained by any given individual model. This fact is not surprising considering that we know that functional and structural brain organization is highly overlapping amongst individuals [43]. Determining what links are more similar across individuals versus those that are shared between individuals is thus an important consideration. In this regard, and consistent with recent work [43] we show in Figure 9 that the regions which have the most variable connections between individuals are those from higher order heteromodal association areas. Interestingly, these are phylogenetically late-developing regions that underwent a disproportionate enlargement during human evolution [29], [43]–[45] as compared to phylogenetically early developing, evolutionarily shared, and unimodal areas, for which we show to have the least functional variability. These finding suggest that the individualizing aspect of the connectotype phenomena is highly dependent on these systems. Thus, these networks are potential targets for tailoring investigations aimed at characterizing individual differences [43] in both research and clinical settings.

### Considerations for Model-Based Connectotyping

We note that our model-based connectivity maps were derived from four ROI atlases, all of which were based on histological studies done in macaque and deformed to fit homologous areas on the human brain. Some atlases performed better than others (see Figure S3 in File S1, median *R*-values are LVE: 0.824, Markov: 0.813, FVE: 0.794, and Paxinos: 0.788, in decreasing order), suggesting that some areal demarcations may be more sensitive to capturing individual differences in connectivity. With that said our results do not appear to be driven by relative number of ROIs.

A potential cofound of our current approach may be that individual differences in anatomy are responsible for the unique models across individuals. To address this concern individualized ROIs were created that account for each subject’s individual anatomy. Interestingly, individualized ROIs had a positive effect in discriminating self *versus* others, as indicated by the increased average correlation coefficient between the predicted and measured bold activity. Furthermore, individualized ROIs left the estimated shared variance in the group unchanged. This indicates that the identified features for each subject are not driven by anatomical differences and highlights that going forward with this approach it might be best to use surface registered regions of interest.

We note that other confounds of human data might also be contributing to the ability to detect a connectotype in individuals. For example, systematic “free thinking” during the resting state, or synchronous movement patterns across time or scan days could lead to a false impression of the data. In accordance with this potential, we have shown that the predictive power and the other properties of this method are also observed on macaque data, whereby animals are lightly anesthetised with little to no movement. While a systematic comparison between the human and macaque connectomes is out of the scope of this manuscript these results highlight the robustness of the technique and also highlight potential benefits to future translational and mechanistic studies [21].

As noted above, in recent years it has been recognized that motion artifacts are one of the biggest challenges facing rs-fcMRI. Given that children and certain patient populations tend to move more than healthy adult subjects, analyses often take extreme precautions by removing frames where even the slightest movement is detected [19], [20]. Prior work has suggested that the removal of frames that contain movement artifact (motion censoring) is sufficient to reduce the noise [19], [20]. However, motion censoring often render entire scans unusable because of the limited data remaining for analyses [19]. Furthermore, these simulations also suggest that after removal of frames up to approximately 4–5 minutes of data remaining, the correlation structure begins to erode [11]. Our approach is able to generate a stable model with as little as 40–60 non-sequential frames (∼2 min); thus, allowing for the most stringent motion censoring techniques while continuing to salvage participants that would otherwise be removed. Similar to these reports, here, we simulate the random removal of frames (as would be the case if a subject was moving) to show that our models are robust to the removal of frames up to a point of <2 minutes remaining. While we also note that there are other methods to correct for motion [19], [46], [47], frame removal is currently quite popular and thus our approach may be an advantage to those who conduct their analyses in this way. This ability is particularly valuable for expensive, difficult to attain, MR data sets of special populations and in publically available databases where the amount of movement in the data is of high concern [19], [48].

Although the current model produces robust individual prediction, it can be improved. For instance, the number of singular values to be truncated is a parameter that it could be optimized. Further, the use of adaptive models, like Kalman filters and the incorporation of historical information into the model (*i.e.* using the actual and historical activity of the functional neighbors to model the activity of each node) will likely improve the approach. Despite these potential improvements, the model was able to precisely characterize a ‘connectotype’ that is tailored to the individual. In addition, we note that smoking, caffeine, alcohol consumption, and/or alertness may contribute to our results regarding the connectotype. However, self-reported data shows no evidence of great heterogeneity on these variables (data shown in Table S1 in File S1 and Table S2 in File S1), which is highly suggestive that these measures alone are not able to classify an individual.

## Conclusions

Considering the vast heterogeneity in both typical and atypical populations, the present approach may allow for significant improvement in our ability to understand individual differences in brain organization. We propose that a better understanding functional brain organization and how it relates to complex behaviors may require investigations starting from the individual [2], [49], [50]. An individual, and the functional circuits that drive their behavioral phenotype, is product of complex brain organization resulting from a combination of unique genetics and environmental exposures. Future work may not only be able to characterize individual connectivity patterns associated with human disease, but also provide individual level targets understanding the influence of environmental and genetic factors. The connectotype may play a role in elucidating these links. While the work requires further development, the findings instills confidence that functional neuroimaging based techniques may have a place in the coming era of personalized medicine.

## Supporting Information

File S1 This file includes 8 supplementary figures and 2 supplementary tables. **Figure S1**, Solutions' and residuals' normalized norm. **Figure S2**, Modeling results across all parcellations. **Figure S3**, Predicting subjects in group H27 (a and b) and group H5(c) using “fresh” data. **Figure S4**, Pinv method, classification power quantified by an ROC curve, group H5. **Figure S5**, Predictions in the group of 5 human subjects (group H5) based on consistent or unique connection weights across the populations. **Figure S6**, Predictions in the group of 27 human subjects (group H27) using pinv, based on consistent or unique connection weights across the populations. **Figure S7**, Predictions in the group of 27 human subjects (group H27) based on consistent or unique connection weights across the populations. **Figure S8**, Individual resting state networks captured by the method on the Markov atlas. **Table S1**, Group H27: gender, age, cigarettes, caffeine, alcohol and sleepiness. **Table S2**, Group H5: gender, age, cigarettes, caffeine, alcohol and sleepiness.
(DOCX)
