# Supplementary material for: Connectotyping: Model Based Fingerprinting of the Functional Connectome
Source: PLoS One. 2014 Nov 11;9(11):e111048. doi: 10.1371/journal.pone.0111048 (PMC4227655; doi:10.1371/journal.pone.0111048)
Supplement: File S1 — This file includes 8 supplementary figures and 2 supplementary tables. Figure S1, Solutions' and residuals' normalized norm. Figure S2, Modeling results across all parcellations. Figure S3, Predicting subjects in group H27 (a and b) and group H5(c) using “fresh” data. Figure S4, Pinv method, classification power quantified by an ROC curve, group H5. Figure S5, Predictions in the group of 5 human subjects (group H5) based on consistent or unique connection weights across the populations. Figure S6, Predictions in the group of 27 human subjects (group H27) using pinv, based on consistent or unique connection weights across the populations. Figure S7, Predictions in the group of 27 human subjects (group H27) based on consistent or unique connection weights across the populations. Figure S8, Individual resting state networks captured by the method on the Markov atlas. Table S1, Group H27: gender, age, cigarettes, caffeine, alcohol and sleepiness. Table S2, Group H5: gender, age, cigarettes, caffeine, alcohol and sleepiness. (DOCX) [file pone.0111048.s001.docx]

Supporting information

# Supplementary Figures:

**
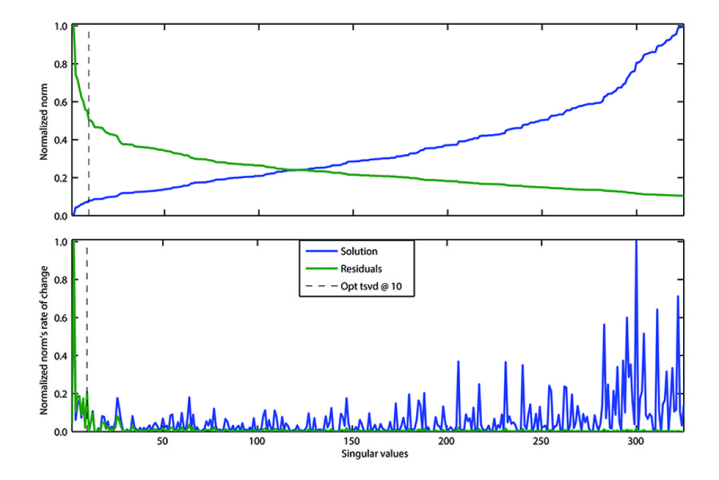
**

Figure S1. Solutions' and residuals' normalized norm. Top panel shows the normalized norm as a function of the singular values used for their calculation. Bottom panel shows their corresponding rate of change. When the truncation is done using 10 singular values, the rate of change for both norms is temporarily stable reaching a local minimum.

**
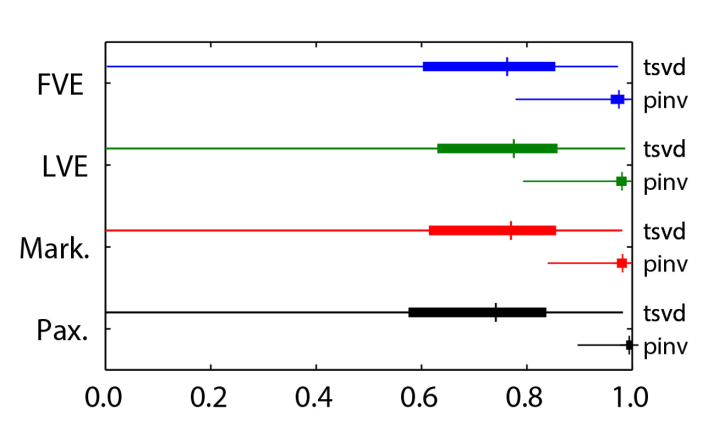
**

Figure S2. Modeling results across all parcellations. The distribution of correlation coefficients between predictions and measured time courses for the 27 subjects, with whole brain regression and movement correction. Pinv and tsvd-based predictions are shown on each panel. Thin lines indicate the range of values, thick lines correspond to the 25 to 75 percentiles and the vertical marker is the median of each distribution. Both methods show a strong ability to predict the timeseries of a given ROI within an individual.


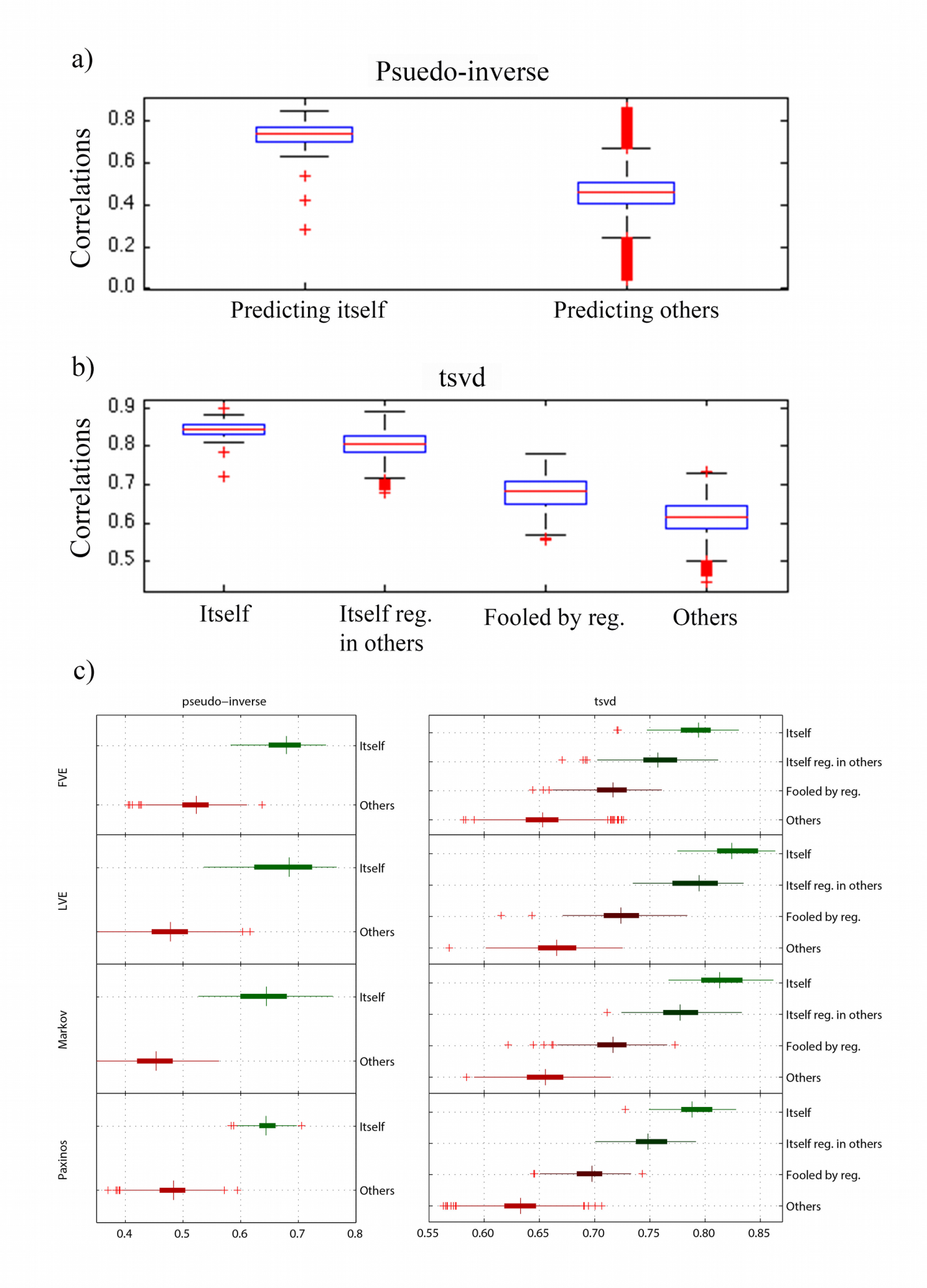


**Figure S3.** Predicting subjects in group H27 (a and b) and group H5(c) using “fresh” data. Panels a and b show the distribution of average correlation coefficients for group H27 predicting itself *versus* predicting others (or their corresponding subgroups) for the FVE parcellation using fresh in-sample data: a) results when the model was obtained using the pseudo inverse; b) results when the model was obtained by tsvd. c) Model predicting same subject on second scan date (*i. e*., subjects in group H5). Distribution of average correlation coefficients for group H5 predicting itself *versus* predicting others. Case "Itself" corresponds to the result when the model, regularization, and prediction were performed on the same subject. "Itself reg. in others" is the case when model and prediction were performed in the same subject, but regularization was performed in a different subject. "Fooled by regularization" comes from the comparison where the regularization and prediction were performed in the same subject, but the model was obtained from a different subject. The remaining combinations are concatenated in the case "Others". In all instances, the model performs more strongly in predicting self versus others; however, a significant amount of variance is accounted for in the “other” cases.


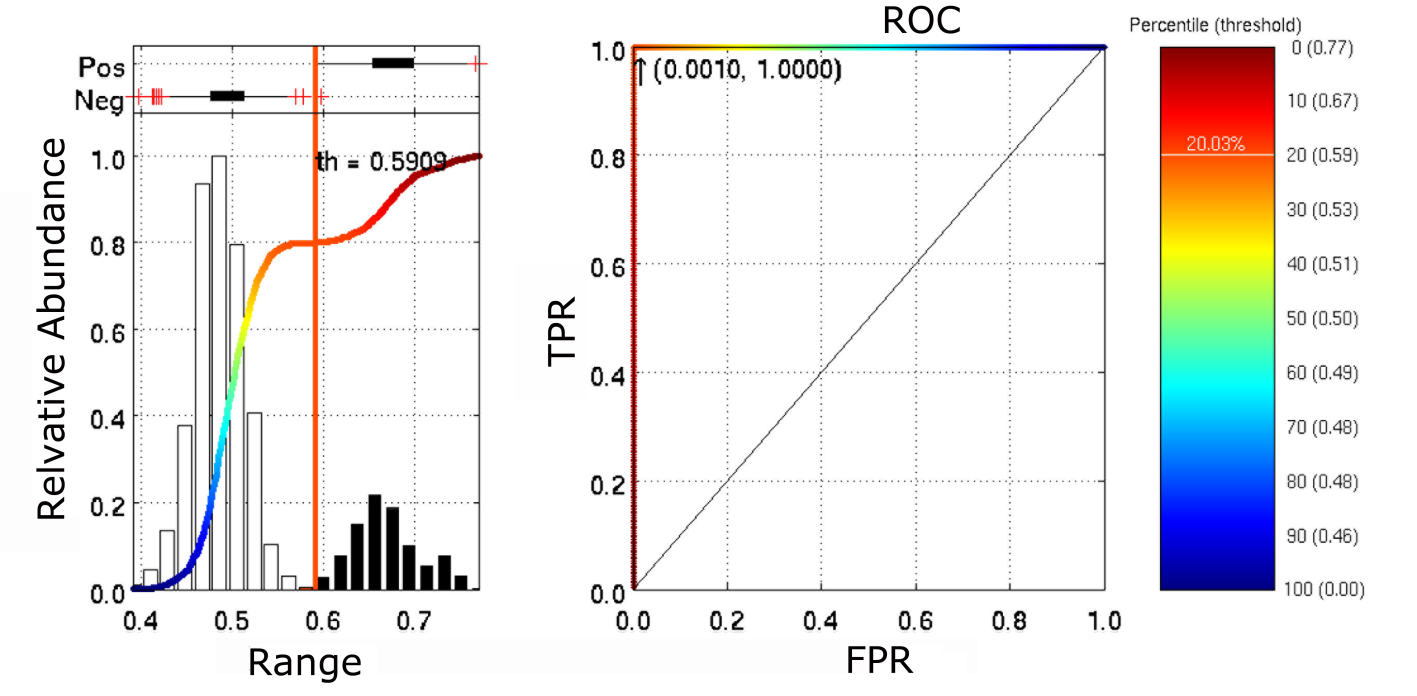


Figure S4. Pinv method, classification power quantified by an ROC curve, group H5. The left panel shows the distributions (top) and the corresponding histograms (bottom) of the correlations of each subject predicting itself (Pos) and predicting others (Neg). The colored line is the cumulative distribution of correlations of the entire data set. The optimal classification is achieved when the threshold is set to 0.5909, which corresponds to the 20.03% of the largest correlation coefficients. This threshold leads to a true positive rate (TPR) of 1 and a false positive rate (FPR) of 0.0010. Analyses were performed using the Markov atlas.


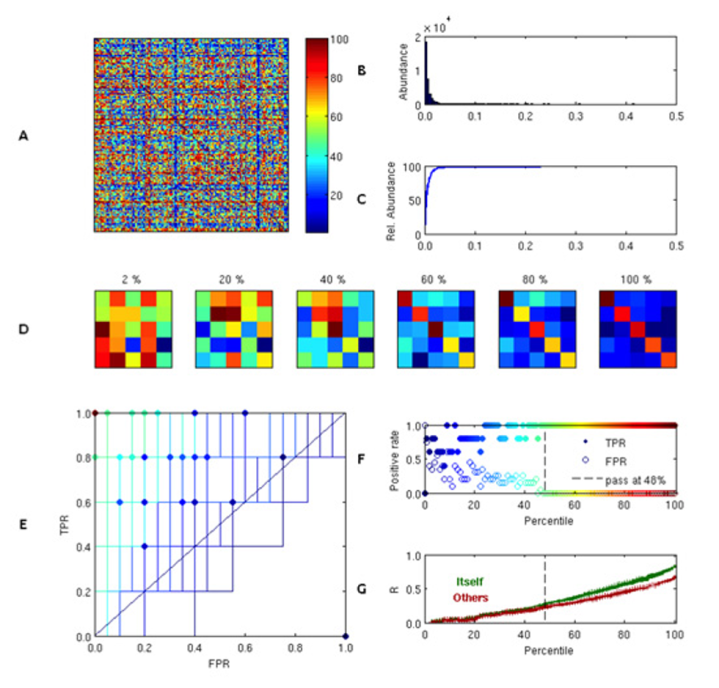


Figure S5. Predictions in the group of 5 human subjects (group H5) based on consistent or unique connection weights across the populations. Predictions are based on the second day scan. A model based pinv connectivity model was calculated for each subject in the H5 group. Then, the variance across participants was calculated for each entry (panel A). Panels B and C show the distribution and cumulative distribution of the coefficient’s variance, respectively. From the cumulative distribution, the connections were grouped into 100 bins with similar variance (presented in panel A). Then, 100 masks were created setting to zero the connections with higher variance and using incrementally the most preserved (lower variance) connections (the first mask has 1% of connections equaling 1 and 99% connections equal to zero, the second mask has 2% of connections equaling ones and 98% of zeros, and so on). The masks were applied to the first day model to predict the second day scan. Panel D shows the correlation coefficient (self-adjusted scale *per* sub-panel) between the predictions and time courses for each one of the subjects in the group H5. The cell *i,j* indicates that the model was obtained in the subject’s *i*first-day scan and that model was used to predict the subject’s *j* second-day time-courses. The sub-panel’s title indicates the percentile of most preserved connections (mask) used in the model. Panel E shows the 100 ROC (lines) and optimal threshold (dots) for each run (percentile). Panel F shows the optimal True Positive Rate (TPR) and False Positive Rate (FPR) as a function of the run. Panel G shows the distributions of correlation coefficients for all the subjects in group H5 predicting itself and predicting others, as a function of run. Dotted line indicates the point at which there is a clear separation between the 2 predictions. All of panels D-F highlight how the predictive nature of the model is dependent on the most variable connections across subjects. The most preserved (lowest variance) connections do little to distinguish between individuals.


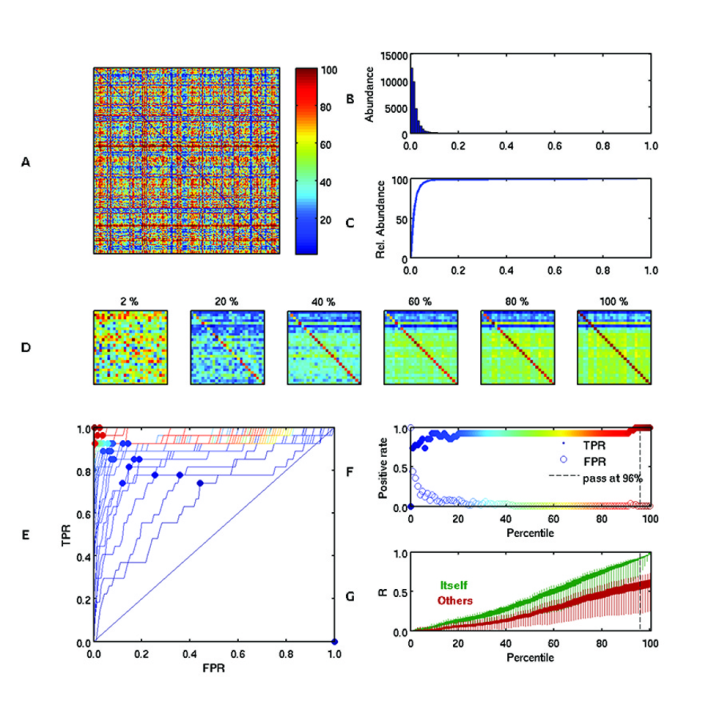


Figure S6. Predictions in the group of 27 human subjects (group H27) using pinv, based on consistent or unique connection weights across the populations. A model based pinv connectivity was calculated for each subject in the H27 group. Then, the variance across participants was calculated for each entry (panel A). Panels B and C show the distribution and cumulative distribution of the coefficient’s variance, respectively. From the cumulative distribution, the connections were grouped into 100 bins with similar variance (presented in panel A). Then, 100 masks were created setting to zero the connections with higher variance and using incrementally the most preserved (lower variance) connections (the first mask has 1% of connections equaling 1 and 99% connections equal to zero, the second mask has 2% of connections equaling ones and 98% of zeros, and so on). The masks were applied to the first day model to predict the second day scan. Panel D shows the correlation coefficient (self-adjusted scale *per* sub-panel) between the predictions and time courses for each one of the subjects in the group H5. The cell *i,j* indicates that the model was obtained in the subject’s *i* first-day scan and that model was used to predict the subject’s *j* second-day time-courses. The sub-panel’s title indicates the percentile of most preserved connections (mask) used in the model. Panel E shows the 100 ROC (lines) and optimal threshold (dots) for each run (percentile). Panel F shows the optimal True Positive Rate (TPR) and False Positive Rate (FPR) as a function of the run. Panel G shows the distributions of correlation coefficients for all the subjects in group H5 predicting itself and predicting others, as a function of run. Dotted line indicates the point at which there is a clear separation between the 2 predictions. All of panels D-F highlight how the predictive nature of the model is dependent on the most variable connections across subjects. The most preserved (lowest variance) connections do little to distinguish between individuals.


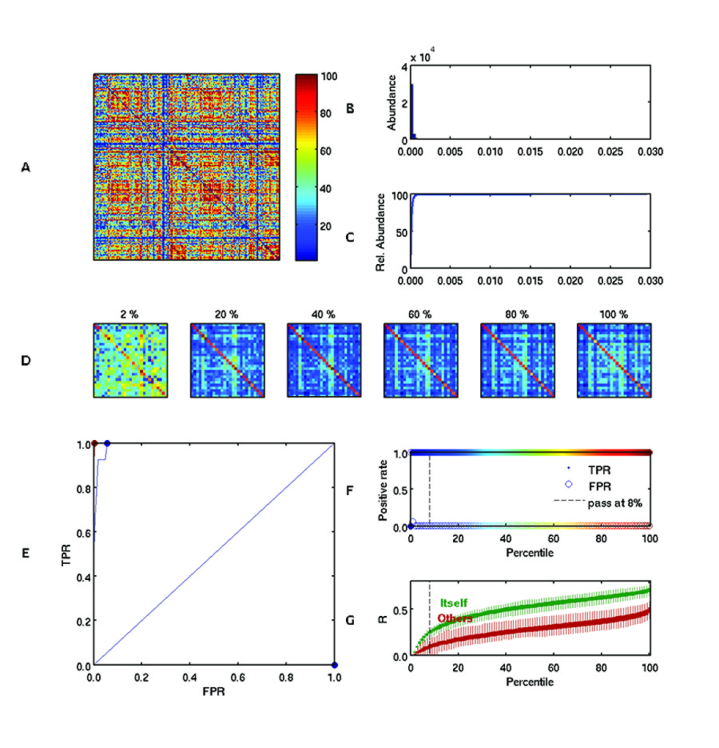


Figure S7. Predictions in the group of 27 human subjects (group H27) based on consistent or unique connection weights across the populations. Predictions are based on the same-day scan. A model based tsvd connectivity model was calculated for each subject in the H27 group. Then, the variance across participants was calculated for each entry (panel A). Panels B and C show the distribution and cumulative distribution of the coefficient’s variance, respectively. From the cumulative distribution, the connections were grouped into 100 bins with similar variance (presented in panel A). Then, 100 masks were created setting to zero the connections with higher variance and using incrementally the most preserved (lower variance) connections (the first mask has 1% of connections equaling 1 and 99% connections equal to zero, the second mask has 2% of connections equaling ones and 98% of zeros, and so on). The masks were applied to the model to predict the scan in fresh data. Panel D shows the correlation coefficient (self-adjusted scale *per* sub-panel) between the predictions and time courses for each one of the subjects in the group H5. The cell *i,j* indicates that the model was obtained in the subject’s *i* first-day scan and that model was used to predict the subject’s *j* “fresh data” time-courses. The sub-panel’s title indicates the percentile of most preserved connections (mask) used in the model. Panel E shows the 100 ROC (lines) and optimal threshold (dots) for each run (percentile). Panel F shows the optimal True Positive Rate (TPR) and False Positive Rate (FPR) as a function of the run. Panel G shows the distributions of correlation coefficients for all the subjects in group H27 predicting itself and predicting others, as a function of run. Dotted line indicates the point at which there is a clear separation between the 2 predictions. All of panels D-F highlight how the predictive nature of the model is dependent on the most variable connections across subjects. The most preserved (lowest variance) connections do little to distinguish between individuals.


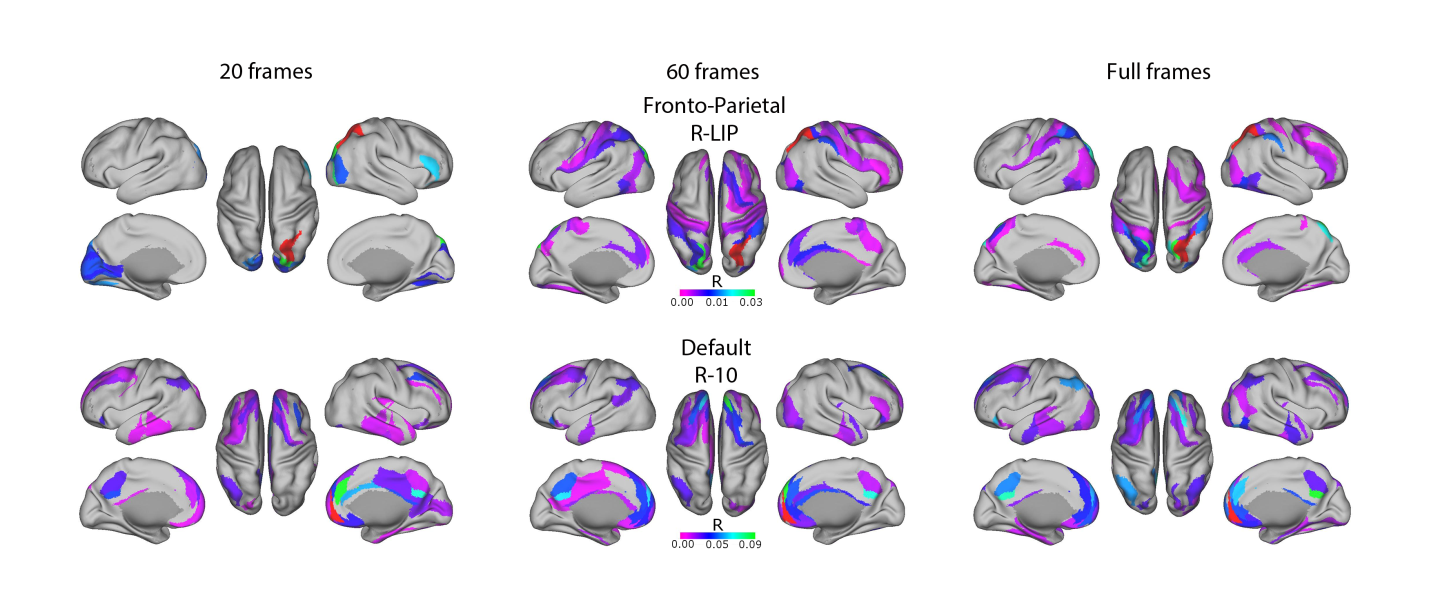


Figure S8. Individual resting state networks captured by the method on the Markov atlas. This figure visualizes networks in a randomly chosen individual subject. In this subject, the default network was well represented even when only 20 frames were used. In contrast, the fronto-parietal network is fairly robust with 60 and full frames, but is not fully represented when using 20 frames.

# Supplementary tables

Supplementary Table S1. Group H27: gender, age, cigarettes, caffeine, alcohol and sleepiness.

| # Participant | Gender | Age | Scan Visit 1 Questionnaire | | | |
| --- | --- | --- | --- | --- | --- | --- |
|  |  |  | Cigs Today | Caffeine Today | Alcohol Yesterday | Sleepiness |
| 1 | M | 29 | 0 | 0.5 | 0 | 3 |
| 2 | F | 27 | 0 | 0 | 0 | N/A |
| 3 | M | 25 | 0 | 0 | 3 | 3 |
| 4 | F | 30 | 0 | 0 | 0 | 6 |
| 5 | M | 27 | 0 | 1 | 0 | 5 |
| 6 | F | 31 | 0 | 2 | 0 | 8 |
| 7 | F | 30 | 0 | 1 | 0 | 4 |
| 8 | M | 27 | 0 | 1 | 0 | 4 |
| 9 | F | 26 | 0 | 0 | 0 | 5 |
| 10 | M | 35 | 3 | 2 | 1 | 5 |
| 11 | F | 24 | 0 | 1 | 0 | 4 |
| 12 | M | 27 | 0 | 0 | 0 | 1 |
| 13 | F | 20 | 0 | 1 | 0 | 8 |
| 14 | F | 31 | 0 | 0 | 0 | 2 |
| 15 | F | 30 | 0 | 1 | 0 | 5 |
| 16 | M | 20 | 0 | 0 | 0 | 6 |
| 17 | F | 22 | 0 | 1 | 0 | 4 |
| 18 | M | 29 | 0 | 0 | 0 | 5 |
| 19 | F | 30 | N/A | N/A | N/A | 4 |
| 20 | M | 22 | 0 | 0 | 0 | 2 |
| 21 | F | 27 | N/A | N/A | N/A | 5 |
| 22 | M | 25 | 0 | 1 | 2 | 2 |
| 23 | F | 19 | N/A | N/A | N/A | 3 |
| 24 | M | 25 | 0 | 2 | 0 | 3 |
| 25 | F | 22 | N/A | N/A | N/A | 5 |
| 26 | F | 22 | N/A | N/A | N/A | 2 |
| 27 | F | 26 | 0 | 1 | 0 | 3 |

Supplementary Table S2. Group H5: gender, age, cigarettes, caffeine, alcohol and sleepiness.

| # | Gender | Age | Scan Visit 1 Questionnaire | | | | Scan Visit 2 Questionnaire | | | |
| --- | --- | --- | --- | --- | --- | --- | --- | --- | --- | --- |
|  |  |  | Cigs Today | Caf. Today | Alc. Yest. | Sleepy | Cigs Today | Caf. Today | Alc. Yest. | Sleepy |
| 5 | M | 27 | 0 | 1 | 0 | 5 | 0 | 1 | 0 | 3 |
| 28 | F | 26 | 0 | 0 | 0 | 5 | 0 | 0 | 0 | 4 |
| 29 | M | 35 | 3 | 2 | 1 | 5 | 4 | 2 | 1 | 4 |
| 30 | F | 20 | 0 | 1 | 0 | 8 | 0 | 1 | 0 | 3 |
| 31 | F | 30 | 0 | 1 | 0 | 5 | 0 | 1 | 0 | 6 |
